# Time-Resolved Whole-Transcriptome Analysis Suggests Candidate Non-Coding RNA Regulatory Networks Associated with PBAN-Induced Pheromone Biosynthesis in *Ostrinia furnacalis*

**DOI:** 10.3390/insects17060652

**Published:** 2026-06-20

**Authors:** Hanbo Zhao, Lei Liu, Bin Yang, Guirong Wang

**Affiliations:** 1 Kunpeng Institute of Modern Agriculture at Foshan, Shenzhen Branch, Guangdong Laboratory of Lingnan Modern Agriculture, Agricultural Genomics Institute at Shenzhen, Chinese Academy of Agricultural Sciences, Shenzhen 518124, China; zhao0b886@nenu.edu.cn; 2 Key Laboratory of Sustainable Forest Ecosystem Management, Ministry of Education, Northeast Forestry University, Harbin 150040, China; oinio@nefu.edu.cn; 3 State Key Laboratory for Biology of Plant Diseases and Insect Pests, Institute of Plant Protection, Chinese Academy of Agricultural Sciences, Beijing 100193, China; byang@ippcaas.cn; 4 Shenzhen Branch, Guangdong Laboratory for Lingnan Modern Agriculture, Synthetic Biology Laboratory of the Ministry of Agriculture and Rural Affairs, Agricultural Genomics Institute at Shenzhen, Chinese Academy of Agricultural Sciences, Shenzhen 518120, China

**Keywords:** PBAN-induced pheromone biosynthesis, non-coding RNA, competing endogenous RNA, whole-transcriptome sequencing, *Ostrinia furnacalis*

## Abstract

The biosynthesis of sex pheromones in lepidopteran pheromone glands is tightly regulated by pheromone biosynthesis-activating neuropeptide (PBAN) signaling; yet the contribution of non-coding RNA-mediated post-transcriptional regulation remains largely unclear. This study aimed to characterize temporal transcriptomic changes, candidate non-coding RNA-mediated regulatory associations, and temporal molecular dynamics underlying transcriptional remodeling after PBAN treatment in Ostrinia furnacalis. First, we performed comprehensive whole-transcriptome sequencing (WTS) on 18 biologically independent samples collected at six time points (0, 20, 40, 60, 90, and 120 min) after PBAN injection. Then, we systematically identified and quantified the dynamic expression patterns of differentially expressed (DE) mRNAs, miRNAs, lncRNAs, and circRNAs in response to PBAN stimulation. By integratively analyzing these multidimensional omics datasets and inferring sequence-based interaction relationships, we inferred a dynamic candidate competing endogenous RNA (ceRNA) like regulatory network. The candidate ceRNA network anchored four core node genes: the PBAN receptor (PBANR), the rate-limiting enzyme acetyl-CoA carboxylase (ACC), and the terminal biosynthetic enzymes desaturase (DES) and fatty acyl-CoA reductase (FAR). The qRT-PCR results further support the temporal expression pattern of key genes during the PBAN response, suggesting that this network can provide a valuable resource for further functional studies.

## Simple Summary

The Asian corn borer (*Ostrinia furnacalis*) is a major lepidopteran pest of maize, and its reproductive success is highly dependent on female-emitted chemical cues that mediate male attraction. Elucidating the biosynthetic and regulatory mechanisms underlying sex-pheromone production is essential for the rational design of environmentally benign pest management strategies. In this study, we characterized the temporal expression profiles of both protein-coding and non-coding RNAs in the pheromone glands of female *O. furnacalis* at multiple time points following treatment with pheromone biosynthesis-activating neuropeptide (PBAN). Our transcriptomic analyses revealed extensive time-dependent changes in the abundance of numerous coding and non-coding transcripts, with the most substantial alterations occurring between 20 and 60 min post-PBAN treatment. Several core genes implicated in signal perception and sex-pheromone biosynthesis exhibited distinct, stage-specific expression trajectories, indicating that pheromone production is likely governed by a coordinated yet sequential regulatory program. Furthermore, we identified multiple candidate regulatory molecules with the potential to modulate the expression of these key genes. Collectively, these results provide a valuable molecular resource for dissecting the regulatory networks that control sex-pheromone production in *O. furnacalis* and highlight putative molecular targets for the development of more precise, sustainable, and environmentally sound pest control strategies.

## 1. Introduction

*Ostrinia furnacalis* (Lepidoptera: Crambidae), commonly known as the Asian corn borer, is a major lepidopteran pest of maize in China, where it is responsible for yield losses of approximately 10–30% [1,2,3]. As in most lepidopteran species, reproductive success in *O. furnacalis* is mediated by female emission of species-specific sex pheromones that attract conspecific males. Owing to the pivotal role of this chemical communication system, the sex pheromone pathway represents an especially suitable and environmentally compatible target for biorational pest management approaches, including mating disruption and mass trapping [4,5,6]. Since the initial characterization in 1980 of its distinctive pheromone components, (Z)- and (E)-12-tetradecenyl acetate, *O. furnacalis* has served as a valuable model to dissect the molecular and biochemical mechanisms underlying pheromone biosynthesis [7]. Studies employing decapitation followed by pheromone biosynthesis-activating neuropeptide (PBAN) injection in moths have demonstrated that the central neuroendocrine peptide PBAN regulates pheromone production by binding to the PBAN receptor (PBANR) and initiating a species-specific intracellular signaling cascade. In *O. furnacalis*, this signaling event modulates sex pheromone titers over a temporal window of 0–120 min [8,9]. PBAN-dependent pathway utilizes Ca^2+^ as the exclusive second messenger to activate calcineurin (CaN), which in turn stimulates acetyl-CoA carboxylase (ACC) [10]. The resulting Ca^2+^/CaN/ACC signaling axis enhances the production of fatty acid precursors that are subsequently converted into the characteristic 53:47 blend of Z/E12-14:OAc through the action of downstream enzymes [11]. The downstream enzymes include multiple gene families, among which the desaturase (DES) and fatty acyl-CoA reductase (FAR) families have been extensively studied [12]. Collectively, these studies have established a robust biochemical and signaling framework for understanding PBAN-mediated pheromone production. However, the post-transcriptional regulatory architecture coordinating PBAN-induced pheromone biosynthesis remains largely unexplored.

In recent years, the classical view of gene regulation has been reshaped by the discovery of non-coding RNAs (ncRNAs), which function as important post-transcriptional regulators in diverse biological processes [13]. In insects, ncRNAs have been implicated in insecticide resistance, innate immunity, development, metamorphosis, and other physiological traits. Among them, microRNAs (miRNAs), typically ~22 nucleotides in length, repress gene expression by binding to complementary sites in target messenger RNAs (mRNAs), leading to mRNA degradation or translational inhibition [14]. In contrast, long non-coding RNAs (lncRNAs) and circular RNAs (circRNAs) can regulate gene expression through the competing endogenous RNA (ceRNA) mechanism [15]. According to this model, lncRNAs and circRNAs act as miRNA sponges or endogenous decoys by competitively binding miRNAs through shared miRNA response elements (MREs), thereby reducing miRNA-mediated repression of target mRNAs [16]. This process gives rise to complex lncRNA/circRNA-miRNA-mRNA regulatory networks [17]. Accumulating evidence suggests that ncRNAs and ceRNA-like networks are involved in multiple insect biological processes, including immune responses, metamorphosis, wing polyphenism, aging, and insecticide resistance [18,19,20,21,22]. However, their roles in the reproductive physiology of agricultural pests, particularly in the regulation of sex pheromone biosynthesis, remain poorly understood. Whether ncRNAs participate in PBAN-induced pheromone production in *O. furnacalis*, and whether they coordinate the expression of core biosynthetic genes through ceRNA interactions remains an open question.

The transition from a resting state to active pheromone production after PBAN stimulation is not a static event but rather a time-dependent physiological cascade involving rapid and ordered changes in gene expression [10,23]. Therefore, analyses restricted to a single time point are insufficient to capture the temporal architecture of PBAN-responsive regulatory networks [12]. Time-course transcriptomic designs provide a more suitable framework for resolving transient, sequential, and stage-specific regulatory events during dynamic biological processes [24]. Accordingly, a comprehensive time-resolved whole-transcriptome analysis is required to investigate potential ceRNA crosstalk among lncRNAs, circRNAs, miRNAs, and mRNAs during PBAN-induced pheromone gland activation. To our knowledge, systematic multi-time-point analyses of PBAN-induced ceRNA regulatory networks in lepidopteran pheromone glands remain lacking.

To characterize temporal transcriptomic changes and candidate non-coding RNA-mediated regulatory associations and temporal molecular dynamics underlying PBAN-induced sex pheromone biosynthesis, we performed comprehensive whole-transcriptome sequencing (WTS) of pheromone glands from female *O. furnacalis*. Recognizing that the onset of active pheromone production represents a tightly time-regulated metabolic cascade, we implemented a high-resolution time-course design, systematically sampling pheromone glands at six successive time points (0, 20, 40, 60, 90, and 120 min) following PBAN injection. This fine-scale temporal framework enabled precise characterization of the rapid and sequential molecular changes occurring throughout the activation process. Through stringent bioinformatic analyses, we globally identified and quantified dynamic expression patterns of differentially expressed (DE) mRNAs, miRNAs, lncRNAs, and circRNAs in response to PBAN stimulation. By integratively analyzing these multidimensional omics datasets and inferring sequence-based interaction relationships, we constructed candidate ceRNA-like regulatory networks, comprising both putative lncRNA–miRNA–mRNA and circRNA–miRNA–mRNA interaction axes, specifically associated with PBAN-mediated sex pheromone biosynthesis in this species. Subsequent functional annotation and network topology analyses enabled the delineation of key candidate protein-coding genes, including PBANR, ACC, DES, and FAR, and critical non-coding RNA regulatory modules that coordinately govern this complex metabolic pathway. Collectively, these findings substantially advance the mechanistic understanding of post-transcriptional regulation in insect sex pheromone biosynthesis and reveal promising molecular targets for agricultural pest control. These candidate ncRNA-mediated regulatory modules may provide molecular entry points for future functional studies and may ultimately inform the development of reproductive interference-based pest management strategies.

## 2. Materials and Methods

*O. furnacalis* populations were reared under standard laboratory conditions: a temperature of 28 °C, relative humidity of 60%, and a photoperiod of 14L:10D. Larvae were fed an artificial diet. To investigate the dynamic regulatory mechanism induced by PBAN, healthy female adults 1 day after eclosion were selected for experiments. To eliminate interference from endogenous PBAN, all females were decapitated and maintained under dark conditions for 24 h. The synthetic PBAN peptide was obtained from Sangon Biotech (Shanghai, China), with the sequence reported by Yao et al. [10]. The peptide was dissolved in double-distilled water and injected into decapitated females at a dose of 10 pmol per individual. The injection was performed between the penultimate and antepenultimate abdominal segments using a microinjection apparatus. Pheromone glands (PGs) were collected at six time points at 0, 20, 40, 60, 90, and 120 min after PBAN injection. The 0 min samples were collected immediately after PBAN injection, without an incubation period, and were used as the baseline group for subsequent time-course comparisons. Each time point included three biological replicates, and each replicate consisted of pooled gland samples from approximately 50 individuals. The collected samples were immediately snap-frozen in liquid nitrogen and then stored in an ultra-low-temperature freezer at −80 °C for subsequent RNA extraction.

Total RNA was extracted from each sample using TRIzol reagent (Invitrogen, Carlsbad, CA, USA). The RNA was treated with DNase I to remove genomic DNA contamination. RNA purity was assessed using a NanoDrop 2000 (Thermo Fisher Scientific, Waltham, MA, USA), and RNA integrity was evaluated with an Agilent 2100 Bioanalyzer (Agilent Technologies, Santa Clara, CA, USA). mRNA, lncRNA, and circRNA libraries were constructed using an RNA library preparation protocol. Specifically, to simultaneously capture transcripts with and without poly-A tails, ribosomal RNA was removed using the Ribo-off rRNA Depletion Kit (Human/Mouse/Rat) (Vazyme Biotech, Nanjing, China). Fragmentation buffer was then added to shear RNA into short fragments. First–strand cDNA was synthesized using random hexamer primers; during second–strand synthesis, dUTP was used in place of dTTP to retain strand–specific information. After end repair, A-tailing, and adapter ligation, the second strand containing dUTP was digested with the USER enzyme. Finally, strand–specific whole–transcriptome libraries were obtained by PCR amplification.

For small RNA sequencing, 18–30 nt RNA fragments were purified by polyacrylamide gel electrophoresis. Specific adapters were ligated to both ends of the fragments, followed by reverse transcription and PCR amplification. The amplified products of 140–160 bp were recovered from the gel to complete library construction. Library quality control was performed using a DNA 1000 Assay Kit (Agilent Technologies, Santa Clara, CA, USA) or a High Sensitivity DNA Assay Kit (Agilent Technologies, Santa Clara, CA, USA). Quantification and pooling were carried out on an ABI StepOnePlus Real-Time PCR System (Life Technologies, Carlsbad, CA, USA). After passing quality control, all libraries were sequenced on the Illumina NovaSeq 6000 platform (Illumina, San Diego, CA, USA). Long RNA libraries were sequenced using a PE150 strategy, and small RNA libraries were sequenced using an SE50 strategy.

Raw sequencing data (raw reads) were first subjected to quality control to remove reads containing adapter sequences, low-quality reads, and reads with an excessively high proportion of unidentified bases. For long-chain RNA (mRNA, lncRNA, and circRNA), the resulting high-quality clean reads were aligned to the Asian corn borer reference genome (GCF_004193835.3) using HISAT2 v2.2.2 with default parameters [25,26]. For small RNA data, raw reads were processed with miRTrace v1.0.1 for filtering and adapter trimming (using *Bombyx mori* as reference), then aligned with Bowtie v1.3.1 to the Rfam database v15.0 to remove contaminating sequences such as rRNA, tRNA, and snRNA [25,27,28].

The aligned long–chain RNA data were used for transcript reconstruction with StringTie v3.0.3 [29]. To identify high-confidence lncRNAs, transcripts shorter than 200 nt and with fewer than two exons were first removed. Next, the coding potential of the remaining transcripts was evaluated using four independent tools: CPAT v3.0.5, CNCI v2, CPC2 v1.0.1, and FEELnc v0.2.1, with default parameters [30,31,32,33]. Only transcripts predicted as non-coding by all four tools were retained as candidate lncRNAs. For each lncRNA gene locus, the longest transcript isoform was retained as the representative transcript. Candidate lncRNAs were further classified into intergenic, antisense, intronic, and sense-overlapping lncRNAs using FEELnc.

Circular RNAs were identified based on back-splice junction signals. Clean reads from rRNA-depleted long RNA libraries were aligned to the reference genome using BWA-MEM v0.7.19. Candidate circRNAs were independently predicted using DCC v0.5.0, CIRI2 v2.0.6, and CIRCexplorer2 v2.3.8 algorithms with default parameters [34,35,36]. To reduce the false-positive rate, only back-splicing events supported by all tools were retained and mapped to their host genes, and the corresponding sequences were extracted for subsequent quantification.

Small RNAs were identified using the miRDeep2 v0.1.3 pipeline. After quality control and contaminant removal, redundant reads were collapsed and aligned to the reference genome with mapper.pl. Known miRNAs were detected by BLASTN homology searches against miRBase [37]. De novo miRNAs were predicted with miRDeep2.pl based on precursor secondary structures, retaining candidates with a miRDeep2 score ≥ 10 and ≥10 mature reads.

Read counts were extracted from the feature-alignment results to construct expression matrices. For mRNAs, lncRNAs, and miRNAs, TPM values were further calculated. For circRNAs, expression levels were normalized using RPM (Reads Per Million mapped reads) based on the number of reads spanning the identified back–splicing junctions (BSJs), and RPM values were used for basic abundance visualization.

Differential expression analysis was performed with DESeq2 v3.22 [38]. For pairwise comparisons between adjacent time points (0 vs. 20, 20 vs. 40, 40 vs. 60, 60 vs. 90, and 90 vs. 120 min), the design formula was ~time_point, and Wald tests were used. RNAs with |log_2_(FoldChange)| > 1 and adjusted *p* < 0.05 were considered significantly differentially expressed. To identify RNAs with temporal changes over the full PBAN response, likelihood ratio tests (LRTs) were used in DESeq2, and RNAs with adjusted *p* < 0.1 were defined as time-responsive. For clustering, transcripts were further filtered for sufficient variation across the six time points (|log_2_(FoldChange)| > 1). Variance-stabilizing transformation (VST) was applied to normalized counts, and mean expression per RNA per time point was calculated from three biological replicates. Time-course clustering was performed with Mfuzz v2.34.0 on z-score-transformed VST matrices, using six clusters for mRNAs and four for lncRNAs, circRNAs, and miRNAs [39]. Cluster centroids were then extracted to evaluate temporal similarities and potential coordinated relationships among mRNA and ncRNA modules using Pearson correlation analysis.

Potential miRNA binding sites on mRNAs, lncRNAs, and circRNAs were predicted using miRanda v3.3a, RNAhybrid v2.1.2, and TargetScan v7.0 [40,41,42]. For mRNAs, 3′ untranslated region sequences were used when available; otherwise, transcript sequences were used. Full-length transcript sequences were used for lncRNAs and circRNAs. Target prediction using miRanda was initially conducted with an alignment score ≥ 100 and a minimum free energy (MFE) ≤ −10 kcal/mol, followed by stricter filtering with an alignment score ≥ 140 and MFE ≤ −20 kcal/mol. For RNAhybrid, the prediction threshold was set to MFE ≤ −25 kcal/mol. Only interactions supported by all three algorithms were retained as candidate miRNA–target pairs. The predicted lncRNA–mRNA, miRNA–mRNA, miRNA–lncRNA, and miRNA-circRNA relationships were used for downstream functional enrichment analysis and candidate ceRNA network construction.

Functional enrichment analysis was performed for mRNA clusters with distinct temporal expression patterns and for predicted protein-coding target genes associated with ncRNAs. For mRNA clusters, genes assigned to each Mfuzz cluster were used for enrichment analysis. For ncRNAs, predicted target genes were collected according to the target prediction results described above, including lncRNA cis/trans target genes and miRNA target mRNAs. Gene Ontology (GO) enrichment and Kyoto Encyclopedia of Genes and Genomes (KEGG) pathway enrichment analyses were performed using the clusterProfiler R package v3.22 [43]. GO terms and KEGG pathways with adjusted *p*-adj < 0.05 were considered significantly enriched.

Candidate pheromone biosynthesis genes, encompassing the PBANR, ACC, DES, and FAR families, were screened using a sequence similarity strategy. Reference sequences for this screening were derived from our previous studies and used in BLASTP searches [44]. To precisely pinpoint functional orthologs within multi-copy families, we extracted the amino acid sequences of the candidate genes and their homologs from closely related lepidopteran species and subjected them to multiple sequence alignment. Subsequently, phylogenetic trees were constructed using the Maximum-Likelihood (ML) method with 1000 bootstrap replicates by IQ-TREE 2 v2.4.0 [45]. By assessing the phylogenetic relatedness of the candidate sequences to experimentally validated Δ14-desaturases and FAR enzymes, the putative DES and FAR candidates were robustly assigned to their respective clades [46,47].

Absolute transcript abundances of core biosynthetic genes (PBANR, ACC, and FAR) and other candidate pheromone biosynthesis genes were quantified as log_2_(TPM + 1). Temporal dynamics of individual genes were shown as line plots with mean ± SEM from three biological replicates. To examine relative transcript composition among the four selected genes, TPM values of the four core genes were summed at each time point to define a total transcript pool, and each gene’s percentage of this pool was calculated and visualized with 100% stacked bar plots.

Based on the ceRNA hypothesis, we systematically constructed multidimensional topological networks for core sex pheromone synthesis genes. Network edges were defined by combining MRE–based targeting evidence with RNA–seq–derived temporal co–expression. The network followed these rules: (1) Core targets: Key pheromone biosynthesis genes (PBANR, ACC, DES, FAR) were set as hubs. (2) Anti-correlated miRNAs: miRNAs targeting these hubs had to show inverse temporal expression (Pearson r < −0.7) relative to the mRNAs and belong to opposite Mfuzz clusters. (3) Co-expressed sponges: Upstream lncRNAs or circRNAs sharing the same MREs had to be positively co-expressed with the target mRNAs. To account for expression level differences, the correlation threshold was r > 0.7 for lncRNA–mRNA pairs and r > 0.3 for circRNA–mRNA pairs to retain low-abundance circular decoys. The final ceRNA interaction matrices were visualized in Cytoscape (v3.9.1) [48].

To validate temporal expression patterns of representative nodes in the candidate ceRNA networks, qRT-PCR was performed on RNA from the same biological batches used for transcriptome sequencing. Based on ceRNA network analysis, two key candidates from each RNA type (miRNA, circRNA, lncRNA) and four related target mRNAs were selected for validation. mRNA, lncRNA, and circRNA were reverse-transcribed with random primers and amplified using TransScript Green One-Step qRT-PCR SuperMix with SYBR Green, while miRNAs were reverse-transcribed using stem-loop or Poly(A) tailing with TransScript^®^ Green miRNA Two-Step qRT-PCR SuperMix (TransGen Biotech, Beijing, China). Divergent primers spanning the back-splice junctions were designed for circRNA validation. *β-actin* was used as the internal reference gene for mRNAs, lncRNAs, and circRNAs, whereas U6 snRNA was used as the internal reference for miRNAs. Three biological replicates were included for each time point. Relative expression levels were calculated using the 2^−ΔΔCt^ method (with 0 min as the calibrator group), and expression trend curves at different time points were plotted [49]. The qRT-PCR results were presented as mean ± standard deviation based on three biological replicates. The temporal expression patterns obtained by qRT-PCR were compared with the RNA-seq expression profiles to assess the consistency of transcriptome-based dynamic trends. The primer sequences used for qRT-PCR are listed in Table S1.

## 3. Results

### 3.1. Sequencing Overview and Sample Reproducibility

**Figure 1:**
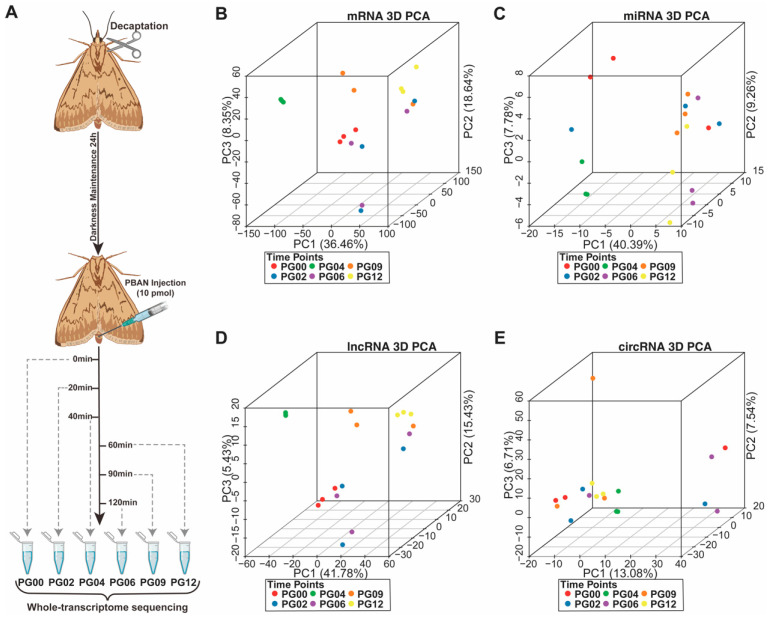
Experimental pipeline and global transcriptomic characterization. (**A**) Schematic workflow of the study. Decapitated female moths were injected with 10 pmols of pheromone biosynthesis-activating neuropeptide (PBAN), and pheromone glands were collected at 0, 20, 40, 60, 90, and 120 min for whole-transcriptome sequencing (WTS), with each time point including three pooled biological replicates, each consisting of pheromone glands from approximately 50 females. (**B**–**E**) Three-dimensional principal component analysis of mRNAs (**B**), miRNAs (**C**), lncRNAs (**D**), and circRNAs (**E**). Colors indicate sampling time points.

To characterize the temporal transcriptional and post-transcriptional responses of pheromone glands to PBAN stimulation in *O. furnacalis*, we performed WTS on 18 biologically independent samples collected at six time points after PBAN injection: 0, 20, 40, 60, 90, and 120 min, with three biological replicates per time point (Figure 1A and Figure S1). Sequencing quality and alignment assessment: Conventional strand-specific RNA-seq generated between 72.02 million and 95.90 million raw reads per sample. After quality control, the uniquely mapped rate to the reference genome remained stable at 58.09% and 64.77%, and the overall mapping rate ranged from 64.26% to 71.96% (Table S2). Small RNA sequencing produced 10.09 million to 16.51 million clean reads. Following adapter removal and length filtering, the number of reads effectively mapped to target regions satisfied the requirements for subsequent quantification (Table S3).

To evaluate variation and reproducibility among treatments, three-dimensional principal component analysis (3D PCA) was performed based on transcripts exhibiting significant fluctuations in the LRT. The PCA model showed that, at the mRNA, miRNA, lncRNA, and circRNA levels, biological replicates at each time point displayed high spatial clustering and followed a clear time-dependent drift trajectory along the PC1 axis (Figure 1B–E), confirming that PBAN stimulation triggered intense and continuous cross-omic transcriptional reprogramming. The raw data have been deposited in the NCBI SRA database under BioProject accession number PRJNA1457832.

### 3.2. Identification and Genomic Features of Non-Coding RNAs

**Figure 2:**
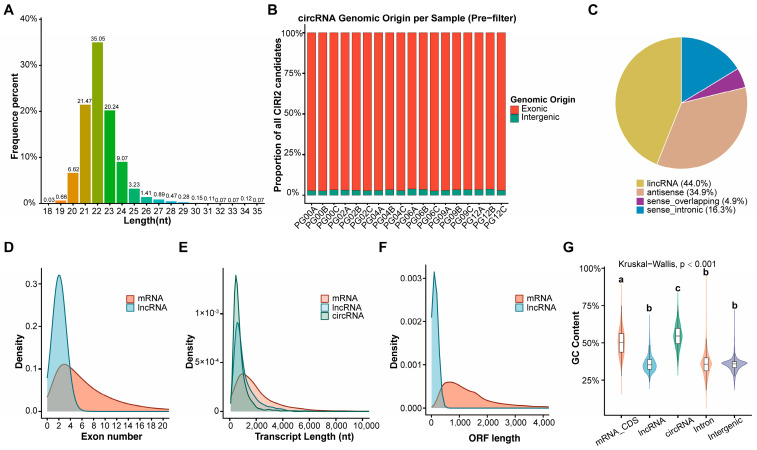
Genomic and structural features of coding and non-coding RNAs. (**A**) Length distribution of identified miRNAs. The different colors of the blocks are utilized solely for visual distinction and do not represent specific biological categories or independent data dimensions. (**B**) Genomic origin composition of circRNA candidates identified by CIRI2 before stringent filtering. (**C**) Genomic classification of identified lncRNAs. Comparison of (**D**) exon number, (**E**) transcript length and (**F**) Open reading frame (ORF) among RNA classes. (**G**) GC content comparison among CDSs, lncRNAs, circRNAs, introns, and intergenic regions. Different letters indicate significant differences based on the Kruskal–Wallis test.

Using a stringent multi-step identification strategy, we systematically annotated miRNAs, circRNAs, and lncRNAs from the whole-transcriptome dataset. For small RNA sequencing, filtered reads were analyzed with the miRDeep2 pipeline and aligned to miRBase, identifying 237 miRNAs (83 known and 154 de novo). Their length distribution showed a typical enrichment between 21 and 24 nt, with 22-nt miRNAs being the most abundant, consistent with Dicer-mediated cleavage (Figure 2A).

We identified 4855 circRNAs across the genome using CIRI2 based on back-splicing junctions, most of which were exonic (Figure 2B). For accurate ceRNA prediction, we stringently selected exonic circRNAs jointly detected by CIRI2, DCC, and CIRCexplorer2, yielding 3339 high-confidence circRNAs (Figure S2A).

By intersecting the results from CPAT, CNCI, CPC2, and FEELnc, we identified 2705 high-confidence lncRNA transcripts, corresponding to 2514 lncRNAs (Figure S2B). Genomically, these lncRNAs were predominantly intergenic (lincRNAs, 44.0%) and antisense (34.9%) (Figure 2C). Compared with protein-coding mRNAs, the identified lncRNAs had fewer exons, shorter transcript lengths, and shorter open reading frames (ORFs) (Figure 2D–F).

Using the Kruskal–Wallis test, we compared GC content across RNA types and genomic regions and found a highly significant global difference (*p* < 0.001). circRNAs had the highest GC content, significantly exceeding that of mRNA coding sequences (CDSs). lncRNAs had relatively low GC content, significantly lower than mRNA CDSs and comparable to introns and intergenic regions (no significant pairwise differences). This base composition pattern matches the typical genomic distribution of non-coding sequences (Figure 2G).

### 3.3. Time-Resolved Differential Expression Reveals a Major Transcriptional Remodeling Window After PBAN Stimulation

To identify the time intervals with the strongest transcriptional changes after PBAN stimulation, pairwise differential expression analysis was performed between adjacent time points using DESeq2. Differentially expressed RNAs were defined using the thresholds |log^2^(FoldChange)| > 1 and adjusted *p* < 0.05. Volcano plots and hierarchical clustering showed that the most pronounced transcriptomic changes occurred between 20 and 60 min after injection (Figures S3–S6). In the 40 min vs. 20 min comparison, a broad decrease in transcript abundance was observed, including 1915 downregulated mRNAs (Figure S3B). In contrast, the subsequent 60 min vs. 40 min comparison showed a marked transcriptional rebound, including 2101 upregulated mRNAs (Figure S3C). Similar temporal changes were also observed in ncRNA datasets, indicating that both coding and non-coding RNAs contributed to the PBAN-responsive transcriptional transition.

To further identify RNAs with significant temporal variation across the entire 0–120 min response period, LRT analysis was performed. RNAs with adjusted *p* < 0.1 and |log^2^(FoldChange)| > 1 across the time course were defined as time-responsive. In total, 6564 differentially expressed mRNAs (DEmRNAs), 985 differentially expressed lncRNAs (DElncRNAs), 2421 differentially expressed circRNAs (DEcircRNAs), and 95 differentially expressed miRNAs (DEmiRNAs) were identified. These results indicate that PBAN stimulation induced extensive time-dependent remodeling at both coding and non-coding RNA levels. The transition from 20 to 60 min, particularly the decrease at 40 min followed by recovery at 60 min, represented a major transcriptional remodeling window during the PBAN response.

### 3.4. Temporal Clustering Reveals Coordinated mRNA and ncRNA Expression Modules

**Figure 3:**
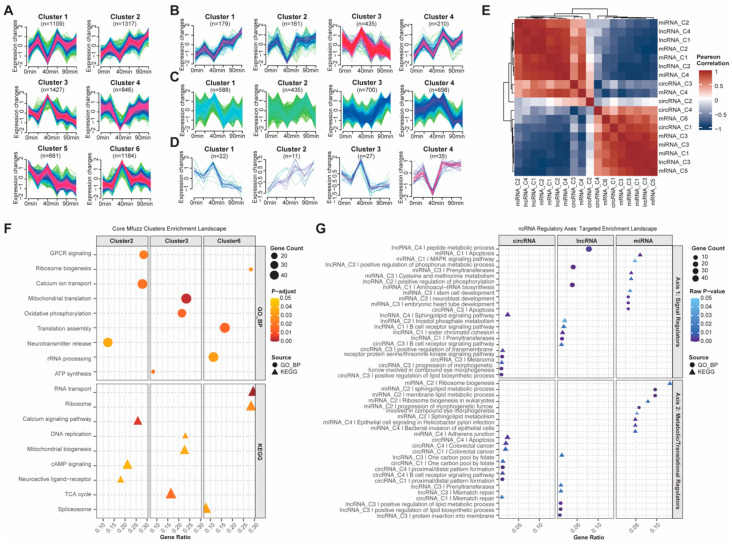
Temporal RNA clusters and functional enrichment. (**A**–**D**) Mfuzz clustering of time-responsive (**A**) mRNAs, (**B**) lncRNAs, (**C**) circRNAs, and (**D**) miRNAs across six PBAN-treated time points. Line colors indicate fuzzy membership values: warm colors represent high membership, whereas cool colors denote low membership. (**E**) Pearson correlation heatmap of cluster centroids across RNA classes. Red and blue indicate positive and negative correlations, respectively. (**F**) GO and KEGG enrichment of representative mRNA clusters. (**G**) Functional enrichment of predicted target genes associated with ncRNA modules correlated with major temporal mRNA clusters. Point size indicates gene count, and color indicates statistical significance.

To classify RNAs according to their temporal expression trajectories, time-responsive RNAs were subjected to Mfuzz soft clustering based on z-score-transformed VST expression values. DEmRNAs were grouped into six clusters, containing 1109, 1317, 1427, 846, 681, and 1184 genes in Clusters 1–6, respectively (Figure 3A). DElncRNAs, DEcircRNAs, and DEmiRNAs were each classified into four clusters. The lncRNA clusters contained 179, 161, 435, and 210 transcripts; the circRNA clusters contained 588, 435, 700, and 698 transcripts; and the miRNA clusters contained 22, 11, 27, and 35 miRNAs, respectively (Figure 3B–D).

To assess temporal coordination among RNA classes, Pearson correlation analysis was performed using cluster centroids. The centroid correlation heatmap revealed several strong positive and negative relationships between mRNA and ncRNA modules (Figure 3E). mRNA Cluster 2 showed strong positive correlations with lncRNA Cluster 1, Cluster 4, and Cluster 2, with correlation coefficients of 0.99, 0.96, and 0.95, respectively. The same mRNA cluster showed strong negative correlations with miRNA Cluster 1 and Cluster 3, with correlation coefficients of −0.95 and −0.90. In addition, mRNA Cluster 3 and Cluster 6 were positively correlated with lncRNA Cluster 3 (r > 0.78) and negatively correlated with miRNA Cluster 4 (r < −0.77). These results suggest that specific lncRNA, circRNA, and miRNA modules exhibit coordinated or opposing temporal patterns relative to key mRNA modules during the PBAN response.

### 3.5. Functional Enrichment of mRNA and ncRNA-Associated Modules

Functional enrichment analysis was performed to characterize the biological processes represented by the major mRNA clusters. Among the six mRNA clusters, Clusters 2, 3, and 6 were associated with biological functions relevant to PBAN signaling, energy metabolism, and translation-related processes (Figure 3F). Genes in Cluster 2 were enriched in pathways related to upstream signal perception and transduction, including G protein-coupled receptors (ko04030) and the calcium signaling pathway (ko04020). GO terms enriched in this cluster included muscle structure development (GO:0061061) and circulatory system process (GO:0003013), suggesting that Cluster 2 contains genes associated with early signal-responsive and transmembrane physiological processes.

Genes in Cluster 3 were enriched in mitochondrial and energy-related biological processes, including mitochondrial gene expression (GO:0140053), mitochondrial translation (GO:0032543), protein targeting (GO:0006605), and protein transmembrane transport (GO:0071806). KEGG enrichment also highlighted mitochondrial biogenesis (ko03029) and DNA replication proteins (ko03032). These enrichment patterns suggest that Cluster 3 may be associated with mitochondrial activity and energy-related transcriptional programs during the PBAN response.

Cluster 6 was associated with the assembly of the translational machinery and was highly enriched in ribosome biogenesis-related processes, including ribonucleoprotein complex biogenesis (GO:0022613), ribosome biogenesis (GO:0042254; ko03009), and transfer RNA biogenesis (ko03016) (Figure 3F). These results indicate that the PBAN response was accompanied by changes in genes related to protein synthesis capacity. In contrast, Clusters 1, 4, and 5 were mainly associated with more general cellular processes, including cytoplasmic translation, ECM–receptor interaction, organic acid metabolism, carboxylic acid metabolism, branched-chain amino acid degradation, and cell-cycle-related processes (Figure S7).

To explore the potential functional relevance of ncRNA-associated modules, predicted target genes of ncRNAs belonging to modules correlated with core mRNA clusters were also subjected to GO and KEGG enrichment analyses. For ncRNA modules associated with mRNA Cluster 2, predicted target genes of lncRNA Cluster 1 were enriched in positive regulation of transmembrane receptor protein serine/threonine kinase signaling pathway (GO:0090100), whereas predicted target genes of lncRNA Cluster 2 were enriched in phosphorylation-related terms, including positive regulation of phosphorylation (GO:0042327) and positive regulation of phosphorus metabolic process (GO:0010562). Predicted target genes of lncRNA Cluster 4 were enriched in peptide metabolic process (GO:0006518) and sphingolipid signaling pathways (ko04071, count = 8). Predicted target genes of miRNA Cluster 1 included genes associated with apoptosis (ko04210) and the signaling pathway (ko04010).

For ncRNA modules associated with mRNA Cluster 3 and Cluster 6, predicted target genes of lncRNA Cluster 3 were enriched in lipid-related and membrane-associated processes, including positive regulation of lipid biosynthetic process (GO:0046889), protein insertion into membrane (GO:0051205), one carbon pool by folate (ko00670), and sphingolipid signaling pathway (ko04071). Predicted target genes associated with circRNA Cluster 1 were also enriched in positive regulation of lipid biosynthetic process (GO:0046889). By contrast, predicted target genes of miRNA Cluster 4 were enriched in mitochondrial membrane organization (GO:0007006) and protein transmembrane import into intracellular organelle (GO:0044743) (Figure 3G). These results indicate that ncRNA modules temporally correlated with major mRNA modules are associated with signaling, lipid metabolism, mitochondrial function, and protein transport-related biological processes. Other ncRNA-associated branches with weaker relevance to the major PBAN-treated time-course-associated modules are shown in Figures S8–S10.

### 3.6. Identification and Temporal Expression Dynamics of Core Pheromone Biosynthesis Genes

**Figure 4:**
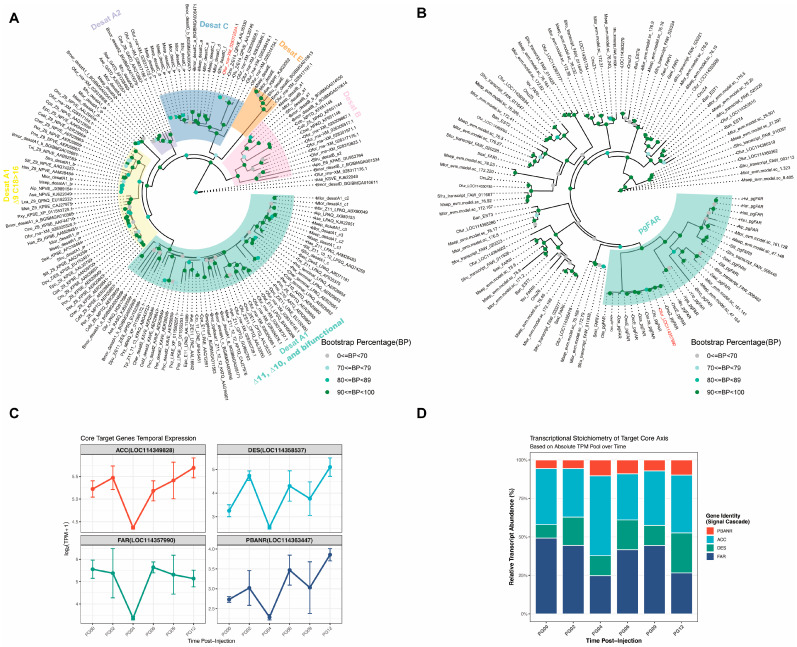
Identification and temporal expression dynamics of core pheromone biosynthesis-related genes. (**A**, **B**) Maximum-likelihood phylogenetic trees of DES (**A**) and FAR (**B**) gene families. Colored blocks denote distinct subfamilies. Candidate DES LOC114358537 and FAR LOC114357990 are highlighted. (**C**) Temporal expression profiles of PBANR, ACC, DES, and FAR after PBAN treatment, shown as mean log^2^(TPM + 1) ± SEM (*n* = 3). Line colors are applied solely for visual distinction. (**D**) Relative transcript composition of the four selected genes across the time course.

To concentrate on genes directly linked to sex pheromone biosynthesis, we systematically screened candidate genes belonging to the *PBANR*, *ACC*, *DES*, and *FAR* gene families within the *O. furnacalis* genome (Figure S11). Because DES and FAR are multi-copy enzyme families, phylogenetic analyzes were performed to identify candidate orthologs associated with pheromone biosynthesis. Maximum-likelihood phylogenetic trees showed that LOC114358537 clustered with experimentally characterized Δ14-desaturases from other lepidopteran insects, whereas LOC114357990 was grouped within a clade containing functionally characterized FAR (Figure 4A,B). Four core genes were selected for further research based on gene family identification, phylogenetic analyses, and expression abundance: *PBANR* (LOC114363447), *ACC* (LOC114349828), *DES* (LOC114358537), and *FAR* (LOC114357990).

The temporal expression profiles of these four genes showed distinct dynamic patterns after PBAN stimulation (Figure 4C). PBANR exhibited a gradual increase in transcript abundance from 0 to 120 min and reached its highest level at 120 min, suggesting sustained expression of the receptor gene during the PBAN response. ACC maintained a relatively stable expression pattern overall, although its absolute TPM value showed a transient decrease at 40 min. When the relative composition of the four-gene transcript pool was examined, ACC accounted for the highest proportion at 40 min, reaching 51.9% of the total TPM of the four core genes (Figure 4D). This indicates that although the total abundance of the core gene pool decreased at 40 min, ACC represented a relatively larger fraction of the remaining core transcripts at this time point.

In contrast, DES and FAR displayed high basal expression levels at 0 min, followed by a pronounced decrease at 40 min and subsequent recovery from 60 to 120 min (Figure 4C). The total TPM of the four core genes decreased from 142.1 at 20 min to 37.8 at 40 min, and this reduction was mainly associated with decreased *DES* and *FAR* transcript abundance. These results indicate that downstream biosynthetic enzyme genes exhibited stronger transient fluctuations than *PBANR* and *ACC* during the mid-phase of the PBAN response. Together, the distinct temporal patterns of *PBANR*, *ACC*, *DES*, and *FAR* suggest that upstream signal perception, precursor supply, and downstream biosynthetic steps may be regulated with different temporal dynamics after PBAN stimulation.

### 3.7. Construction of Candidate ceRNA-like Networks and qRT-PCR Analysis of Representative Nodes

**Figure 5:**
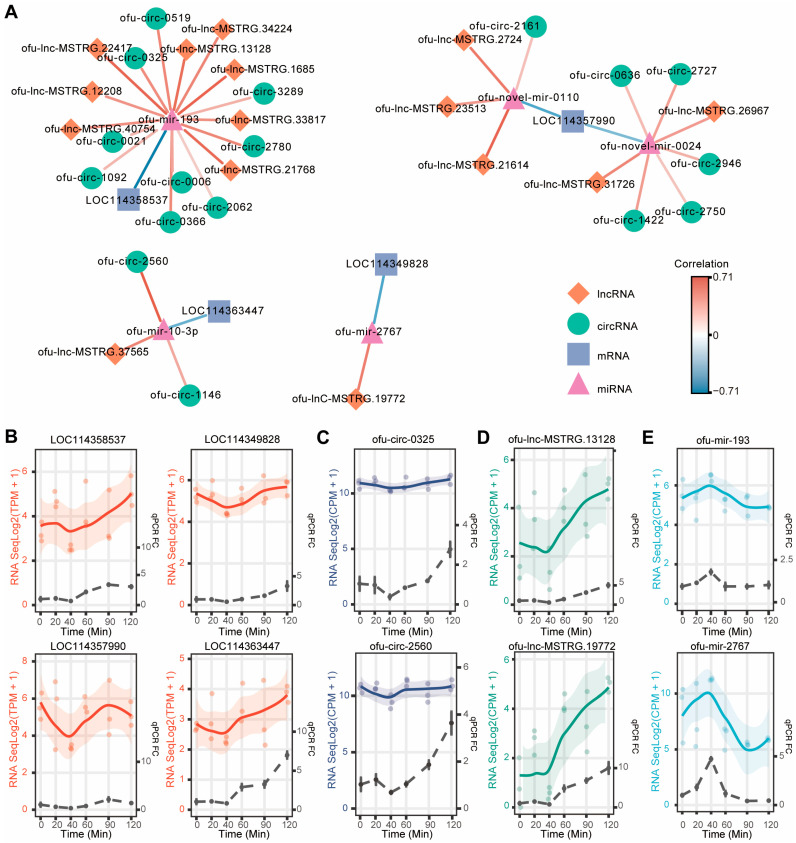
Candidate ceRNA-like subnetworks and qRT-PCR analysis of representative transcripts. (**A**) Cytoscape visualization of candidate subnetworks centered on four core mRNAs: LOC114358537, LOC114349828, LOC114357990, and LOC114363447. Nodes represent RNA biotypes, and edges indicate predicted miRNA–target relationships supported by temporal expression correlations. (**B**–**E**) Temporal expression patterns of representative mRNAs (**B**), circRNAs (**C**), lncRNAs (**D**), and miRNAs (**E**) based on sequencing data and qRT-PCR. Sequencing abundance is shown as log_2_(TPM + 1) or log_2_(CPM + 1), and qRT-PCR values are shown as relative expression calculated by the 2^−ΔΔCt^ method. Line colors denote distinct RNA types.

Based on the ceRNA hypothesis, candidate regulatory networks centered on the four core pheromone biosynthesis-related genes were constructed by integrating sequence-based target prediction and expression correlation. Candidate miRNA–mRNA, miRNA–lncRNA, and miRNA–circRNA interactions were first predicted using miRanda, RNAhybrid, and TargetScan, and only interactions supported by all three algorithms were retained. For network construction, core mRNAs were required to share predicted miRNA response elements with lncRNAs or circRNAs. In addition, miRNAs targeting core mRNAs were required to show a negative temporal correlation with their predicted mRNA targets, whereas lncRNAs or circRNAs sharing the same miRNAs were required to show a positive temporal correlation with the corresponding mRNAs. Using these filtering criteria, candidate ceRNA-like subnetworks were obtained for *PBANR*, *ACC*, *DES*, and *FAR* (Figure 5A).

The DES-centered subnetwork showed the highest network complexity among the four core genes. This module predicted that ofu-miR-193 targets *DES* (LOC114358537), with several lncRNAs and circRNAs, such as ofu-lnc-MSTRG.13128 and ofu-circ-0325, exhibiting predicted binding interactions with the same miRNA. The *FAR*-centered subnetwork was additionally linked to multiple ncRNA candidates. *FAR* (LOC114357990) was predicted to be associated with ofu-novel-miR-0024 and related lncRNA/circRNA nodes, including ofu-lnc-MSTRG.21614 and several circRNAs. By comparison, the PBANR- and *ACC*-centered subnetworks were more compact. *PBANR* (LOC114363447) was mainly associated with ofu-miR-10-3p and selected circRNA candidates such as ofu-circ-1146, whereas *ACC* (LOC114349828) formed a smaller candidate regulatory axis involving ofu-miR-2767 and ofu-lnc-MSTRG.19772.

To validate temporal expression trends from high-throughput sequencing, representative mRNAs, lncRNAs, circRNAs, and miRNAs from candidate ceRNA networks were analyzed by qRT-PCR at six *PBAN* response time points (Figure 5B–E). qRT-PCR largely confirmed the sequencing-based trends: selected mRNAs, lncRNAs, and circRNAs decreased and then recovered between 20 and 60 min, while selected miRNAs showed the opposite pattern, increasing as their target mRNAs declined. Although circRNAs had relatively low expression, their qRT-PCR profiles broadly matched the RNA-seq patterns.

## 4. Discussion

PBAN is a key activator of pheromone biosynthesis in lepidopteran pheromone glands, but the temporal coordination between coding and non-coding transcriptomes in this process remains unclear [8]. In this study, we implemented a whole-transcriptome time-series design encompassing six post-stimulation time points to characterize the dynamics of mRNAs, miRNAs, lncRNAs, and circRNAs following PBAN treatment in *O. furnacalis* (Figure S1). The temporal separation of samples in PCA and the expected structural features of the identified ncRNAs support the overall consistency of the dataset and the reliability of subsequent descriptive analyses (Figure 1 and Figure 2). Leveraging this resource, we demonstrate that PBAN stimulation elicits a broad, stage-specific transcriptomic response, manifested as coordinated alterations in protein-coding genes and multiple classes of ncRNAs. These findings establish a temporal framework for elucidating how PBAN-responsive regulatory modules may govern the dynamic regulation of pheromone biosynthesis, and they further enable the inference of candidate ceRNA networks centered on core biosynthetic genes.

Adjacent time-point comparisons showed that 20–60 min was the main transition phase in the PBAN response (Figures S3–S6). Relative to 20 min, many transcripts decreased at 40 min and then increased again at 60 min. Similar transient or pulse-dependent transcriptional responses occur in other hormone-regulated systems, such as ecdysone-responsive gene cascades in insects and norepinephrine-induced transient down-regulation with recovery of β3-adrenoceptor expression in brown fat cells [50,51,52]. Although biologically distinct from PBAN signaling in pheromone glands, these systems demonstrate that hormone-responsive transcriptional programs can be transient and non-monotonic.

Temporal clustering showed that distinct functional modules contributed to different layers of the PBAN response (Figure 3). Among major mRNA clusters, Cluster 2 was enriched for upstream signal perception and transduction pathways, including G protein-coupled receptors and calcium signaling; Cluster 3 was mainly associated with mitochondrial activity, protein targeting, and transmembrane transport; and Cluster 6 was enriched in ribosome and tRNA biogenesis, indicating altered protein synthesis capacity. Thus, PBAN-time-course-associated mRNA modules span multiple functional layers, including signal perception, mitochondrial activity, membrane/protein transport, and translation-related processes [10,12,53,54]. However, some enriched GO terms, particularly those related to mitochondrial activity, translation, membrane/protein transport, and other broad cellular processes, may reflect general cellular responses to PBAN treatment rather than pheromone-specific regulatory events.

The ncRNA modules correlated with these mRNA clusters showed partially overlapping functional associations via their predicted target genes. For Cluster 2, positively correlated lncRNA modules were linked to target genes enriched in receptor signaling, phosphorylation, peptide metabolism, and sphingolipid signaling, while negatively correlated miRNA modules were associated with MAPK signaling and apoptosis-related pathways.

For mRNA Clusters 3 and 6, positively correlated lncRNA/circRNA modules were linked to predicted targets enriched in lipid biosynthetic, membrane insertion, and sphingolipid signaling, whereas the negatively correlated miRNA module was associated with mitochondrial membrane organization and protein transmembrane import. Overall, predicted targets of temporally correlated ncRNA modules were enriched in terms related to signaling, lipid metabolism, mitochondrial function, and transport, suggesting that these ncRNAs may be linked to PBAN-responsive cellular programs. These associations are still computational and require functional validation, but they suggest that ncRNA modules temporally coupled with major mRNA clusters may be involved in signaling, lipid metabolism, mitochondrial, and transport processes during PBAN-induced pheromone gland activation [23,55,56,57,58].

The opposing temporal patterns among mRNA, miRNA, lncRNA, and circRNA modules are consistent with a ceRNA-like regulatory model. Under the ceRNA hypothesis, RNAs sharing miRNA response elements can affect each other by competing for miRNA binding [15]. In this study, several lncRNA and circRNA modules were positively correlated with major mRNA modules, whereas specific miRNA modules displayed inverse temporal patterns, supporting the construction of candidate lncRNA/circRNA–miRNA–mRNA interaction networks. However, endogenous miRNA sponge activity is highly context-dependent, influenced by RNA abundance, miRNA availability, binding-site number and affinity, and subcellular co-localization. [15,17]. Thus, the networks proposed here should be viewed as candidate ceRNA-like regulatory frameworks rather than experimentally confirmed sponge mechanisms.

To more directly link the global transcriptomic response with pheromone biosynthesis, we focused on four core genes representing key pathways: *PBANR* (signal perception), *ACC* (fatty-acid precursor supply), *DES* (desaturation), and *FAR* (reduction). Phylogenetic and sequence analyses supported LOC114358537 as a Δ14-desaturase candidate and LOC114357990 as a *FAR* candidate (Figure 4A,B). *PBANR* expression gradually increased after PBAN stimulation, whereas *ACC* stayed largely stable, aside from a transient TPM decrease at 40 min. In contrast, *DES* and *FAR* showed a sharp drop at 40 min and then recovered from 60 to 120 min (Figure 4C,D). This tier-specific pattern indicates that PBAN-induced pheromone biosynthesis does not involve uniform transcriptional activation of all pathway components. Similar stepwise PBAN regulation has been observed in other moths, including *Conogethes punctiferalis* and *Heliothis virescens* [23,59]. Thus, upstream signal perception, precursor supply, and downstream biosynthetic conversion are temporally uncoupled at the transcript level.

Based on this rationale, we built candidate *PBANR*-, *ACC*-, *DES*-, and *FAR*-centered ceRNA-like subnetworks by integrating sequence-based target prediction with temporal expression correlations (Figure 5). The *DES*- and *FAR*-centered subnetworks contained more ncRNA nodes than the *PBANR*- and *ACC*-centered subnetworks, indicating that downstream biosynthetic genes may be embedded in relatively complex putative post-transcriptional regulatory architectures. Several miRNAs, including ofu-miR-193, ofu-miR-10-3p, ofu-miR-2767, and two newly predicted miRNAs, were retained as candidate regulators based on target prediction and inverse temporal expression patterns. Some of these miRNAs have been functionally implicated in other insects or animals.

For example, miR-193 is involved in pigmentation regulation in Lepidoptera, and mammalian miR-193a-5p regulates fatty acid desaturase 1 in lipid metabolism [60,61]. miR-10-3p and miR-2767 have also been detected in insect immune or stress-related small RNA datasets [62,63]. In addition, the miR-10 family is an important regulator of fatty acid metabolism in animals [64,65]. qRT-PCR validation supported the temporal expression patterns of representative mRNAs, miRNAs, lncRNAs, and circRNAs in the candidate networks. Overall, selected mRNAs, lncRNAs, and circRNAs showed decrease-and-recovery patterns consistent with the RNA-seq profiles, whereas selected miRNAs generally displayed opposite trends to their predicted target mRNAs. These findings support the reliability of the transcriptome-based dynamic patterns and highlight these ncRNAs as priority candidates for future validation.

This research offers a time-resolved whole-transcriptome resource for PBAN-responsive pheromone glands in *O. furnacalis* and identifies coordinated coding and non-coding RNA modules associated with sex pheromone biosynthesis. These results suggest that upstream signal perception, precursor supply, and downstream biosynthetic conversion display distinct temporal expression patterns after PBAN stimulation. The candidate ceRNA networks centered on these core genes provide a set of prioritized regulatory axes for future investigation. Nevertheless, the present evidence is mainly based on sequence-based target prediction, expression correlation, and qRT-PCR validation of temporal trends.

Accordingly, the proposed networks should be regarded as hypothesis-generating rather than as mechanistically validated regulatory architectures. The candidate ceRNA networks were inferred using a conservative, multi-layer filtering framework that integrated computational target prediction, the presence of shared MREs, consistency in expression-direction, and correlation-based thresholds. As such, they were designed to prioritize testable hypotheses for subsequent functional interrogation rather than to delineate definitive regulatory interactions. Moreover, although this study covered the reported 0–120 min PBAN-responsive period in *O. furnacalis*, denser early and later sampling will be needed to fully resolve PBAN-associated transcriptomic kinetics. Future functional studies with expanded temporal sampling and functional validation will be necessary to determine whether the candidate miRNAs, lncRNAs, and circRNAs directly regulate PBAN-induced pheromone biosynthesis and whether these regulatory modules can be exploited as molecular entry points for reproductive interference-based pest management.

## 5. Conclusions

This study provides a time-resolved, whole-transcriptome characterization of coding and non-coding RNA dynamics in the pheromone glands of *O. furnacalis* following stimulation with PBAN. The data demonstrate extensive temporal reprogramming of mRNAs, miRNAs, lncRNAs, and circRNAs, with the most pronounced transcriptional alterations occurring within the 20–60 min post-stimulation interval. Key pheromone biosynthesis-associated genes, including *PBANR*, *ACC*, *DES*, and *FAR*, exhibited distinct temporal expression trajectories, indicating that signal perception, precursor provision, and terminal biosynthetic conversion are likely regulated in a coordinated yet sequential manner during pheromone gland activation. Through integration of expression correlation analyses with predicted miRNA–mRNA interactions, we further delineated candidate ceRNA-like regulatory networks centered on these core biosynthetic genes. qRT-PCR assays performed on representative transcripts corroborated the temporal expression patterns inferred from the sequencing datasets. Collectively, this work provides a comprehensive transcriptomic resource and a prioritized set of candidate regulatory molecules for subsequent functional dissection of pheromone biosynthesis in *O. furnacalis*. Moreover, these insights may inform the rational design of more precise and environmentally sustainable strategies for the management of this agricultural pest.

## Data Availability

The sequencing data generated in this study have been deposited in the NCBI Sequence Read Archive under BioProject accession number PRJNA1457832. The processed data and supporting results generated during this study are included in this article and its Supplementary Materials. Additional information is available from the corresponding author upon reasonable request.

## Supplementary Materials

The following supporting information can be downloaded at: https://www.mdpi.com/article/10.3390/insects17060652/s1, Figure S1: Whole Transcriptome Sequencing (WTS) process flow to construct the ceRNA network; Figure S2: Identification of high-confidence circRNAs and lncRNAs; Figure S3: Statistical results and clustering analysis of differentially expressed mRNAs at sequential time points after PBAN injection; Figure S4: Statistical results and clustering analysis of differentially expressed lncRNAs at sequential time points after PBAN injection; Figure S5: Statistical results and clustering analysis of differentially expressed circRNAs at sequential time points after PBAN injection; Figure S6: Statistical results and clustering analysis of differentially expressed miRNAs at sequential time points after PBAN injection; Figure S7: Functional enrichment landscape of mRNA Mfuzz clusters; Figure S8: Functional enrichment landscape of target genes for lncRNA Mfuzz clusters; Figure S9: Functional enrichment landscape of target genes for circRNA Mfuzz clusters; Figure S10: Functional enrichment landscape of target genes for miRNA Mfuzz clusters; Figure S11: Temporal expression dynamics and absolute abundance of putative pheromone biosynthesis gene families; Table S1: All primers used in this study; Table S2: RNA-seq clean data quality inspection analysis; Table S3: miRNA-seq clean data quality inspection analysis.

## Abbreviations

The following abbreviations are used in this manuscript:

PBAN	Pheromone biosynthesis-activating neuropeptide
WTS	Whole-transcriptome sequencing
DE	Differentially expressed
ceRNA	Competing endogenous RNA
PBANR	PBAN receptor
ACC	acetyl-CoA carboxylase
DES	Desaturase
FAR	Fatty acyl-CoA reductase
PG	Pheromone gland
CaN	Calcineurin
ncRNA	Non-coding RNA
miRNA	MicroRNA
mRNA	Messenger RNA
lncRNA	Long non-coding RNA
circRNA	Circular RNA
MRE	MicroRNA response element
BSJ	Back-splicing junction
LRT	Likelihood ratio test
minimum free energy	MFE
VST	Variance-stabilizing transformation
GO	Gene Ontology
KEGG	Kyoto Encyclopedia of Genes and Genomes
ML	Maximum likelihood
3D PCA	Three-dimensional principal component analysis
ORF	Open reading frame
CDS	Coding sequence
DEmRNA	Differentially expressed mRNA
DElncRNA	Differentially expressed lncRNA
DEcircRNA	Differentially expressed circRNA
DEmiRNA	Differentially expressed miRNA
SEM	Standard error of the mean
SD	Standard deviation
