# Supplementary material for: Time-Resolved Whole-Transcriptome Analysis Suggests Candidate Non-Coding RNA Regulatory Networks Associated with PBAN-Induced Pheromone Biosynthesis in Ostrinia furnacalis
Source: Insects. 2026 Jun 20;17(6):652. doi: 10.3390/insects17060652 (PMC13300231; doi:10.3390/insects17060652)
Supplement: Supplementary file 1 [file insects-17-00652-s001.zip › insects-4351658-supplementary.pdf]

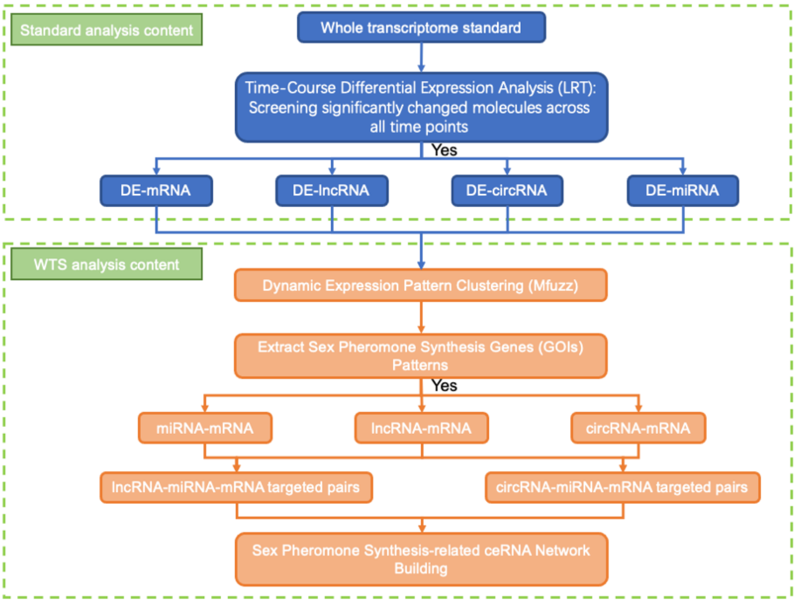

Figure S1. Whole Transcriptome Sequencing (WTS) process flow to construct the ceRNA network.

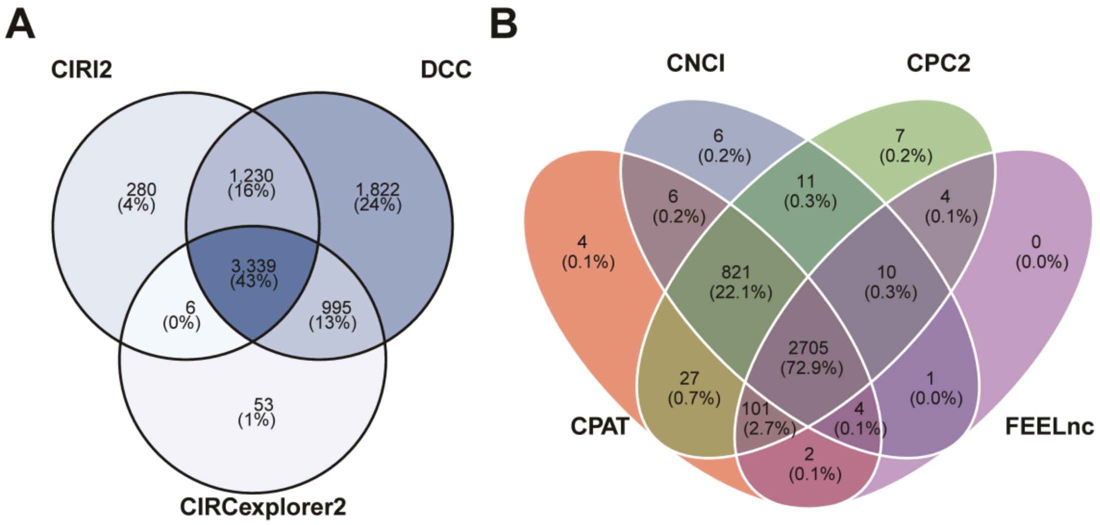

Figure S2. Identification of high-confidence circRNAs and lncRNAs. (A) Venn diagram showing the overlap of circRNA candidates predicted by three independent circRNA detection tools: CIRI2, DCC, and CIRCexplorer2. CircRNAs supported by all three tools were retained as high-confidence circRNA candidates for downstream analysis, yielding 3,339 circRNAs. (B) Venn diagram showing the overlap of candidate lncRNA transcripts predicted as non-coding by four coding-potential assessment tools: CNCI, CPC2, CPAT, and FEELnc. Transcripts consistently classified as non-coding by all four tools were retained as high-confidence lncRNA candidates, yielding 2,705 lncRNA transcripts. Numbers and percentages indicate the size and relative proportion of each intersecting subset.

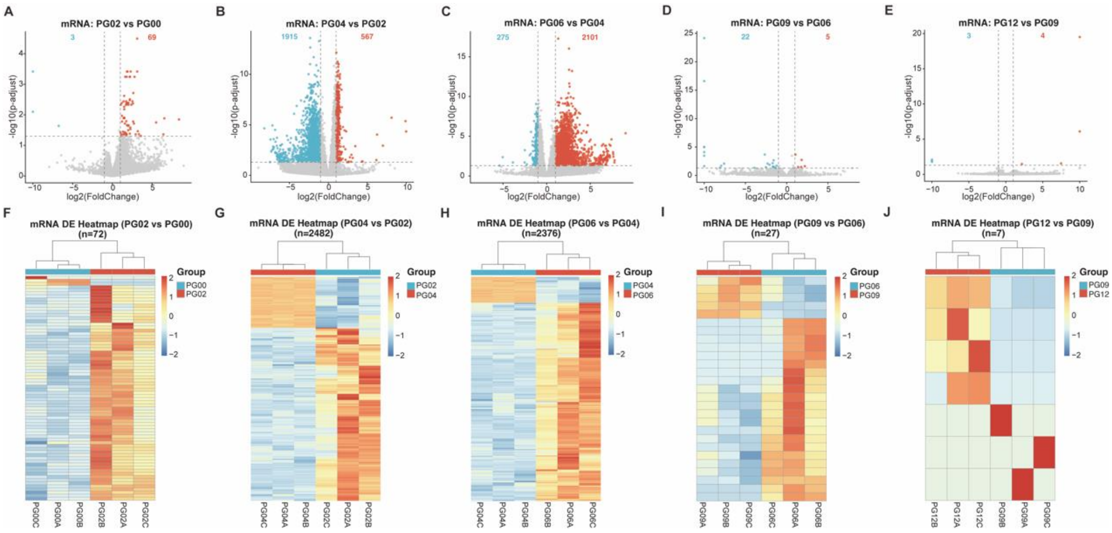

Figure S3. Statistical results and clustering analysis of differentially expressed mRNAs at sequential time points after PBAN injection. Volcano plots of differentially expressed mRNAs for (A) 20 min vs 0 min, (B) 40 min vs 20 min, (C) 60 min vs 40 min, (D) 90 min vs 60 min, and (E) 120 min vs 90 min. Red and blue dots represent significantly up-regulated and down-regulated transcripts, respectively (screening criteria: P-adj < 0.05 and |log2(FoldChange)| > 1). Hierarchical clustering analysis of differentially expressed mRNAs for (F) 20 min vs 0 min, (G) 40 min vs 20 min, (H) 60 min vs 40 min, (I) 90 min vs 60 min, and (J) 120 min vs 90 min.

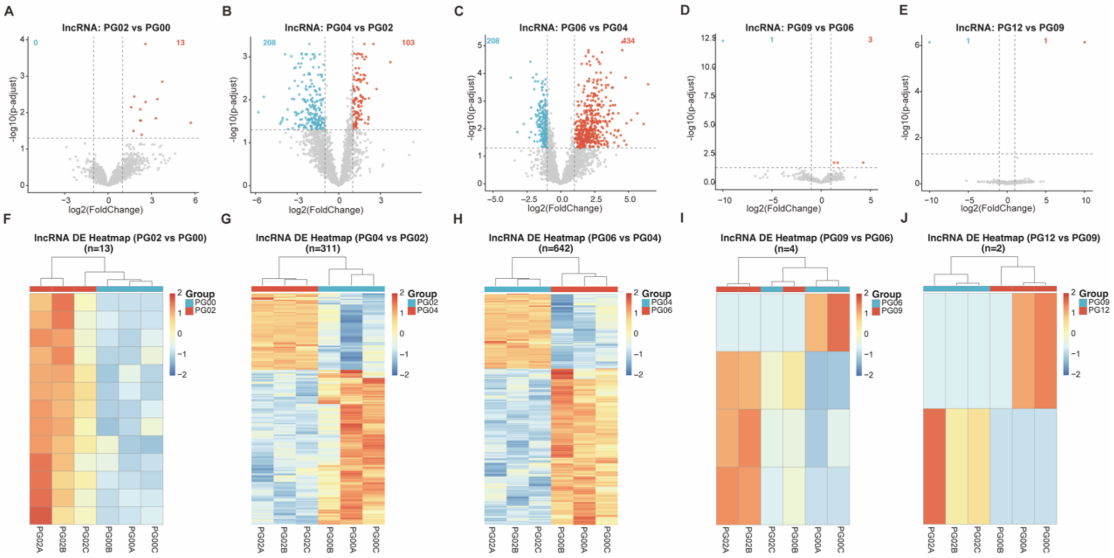

Figure S4. Statistical results and clustering analysis of differentially expressed lncRNAs at sequential time points after PBAN injection. (A–E) Volcano plots of differentially expressed lncRNAs for (A) 20 min vs 0 min, (B) 40 min vs 20 min, (C) 60 min vs 40 min, (D) 90 min vs 60 min, and (E) 120 min vs 90 min. Red and blue dots represent significantly up-regulated and down-regulated transcripts, respectively (screening criteria: P-adj < 0.05 and |log2(FoldChange)| > 1). Hierarchical clustering analysis of differentially expressed lncRNAs for (F) 20 min vs 0 min, (G) 40 min vs 20 min, (H) 60 min vs 40 min, (I) 90 min vs 60 min, and (J) 120 min vs 90 min.

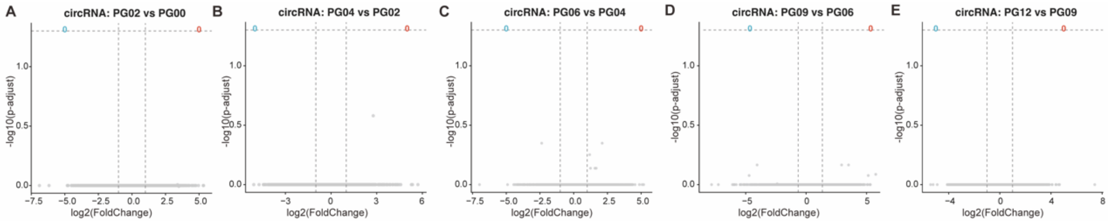

Figure S5. Statistical results and clustering analysis of differentially expressed circRNAs at sequential time points after PBAN injection. Volcano plots of differentially expressed circRNAs for (A) 20 min vs 0 min, (B) 40 min vs 20 min, (C) 60 min vs 40 min, (D) 90 min vs 60 min, and (E) 120 min vs 90 min.

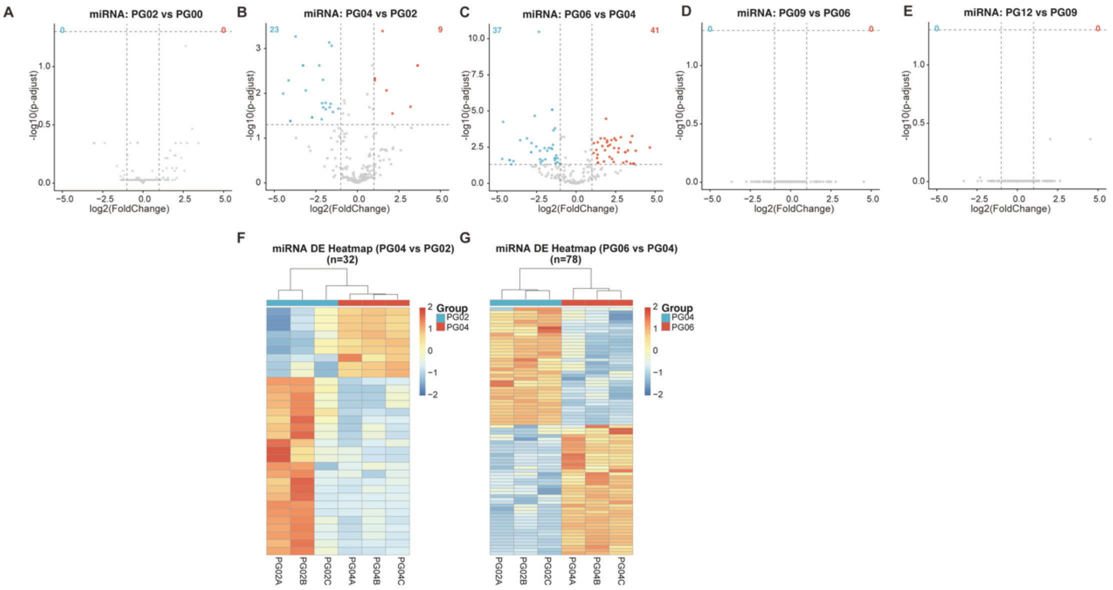

Figure S6. Statistical results and clustering analysis of differentially expressed miRNAs at sequential time points after PBAN injection. Volcano plots of differentially expressed miRNAs for (A) 20 min vs 0 min, (B) 40 min vs 20 min, (C) 60 min vs 40 min, (D) 90 min vs 60 min, and (E) 120 min vs 90 min. Red and blue dots represent significantly up-regulated and down-regulated transcripts, respectively (screening criteria: P-adj < 0.05 and $|\log2(\text{FoldChange})| > 1$). Hierarchical clustering analysis of differentially expressed miRNAs for (F) 40 min vs 20 min, (G) 60 min vs 40 min.

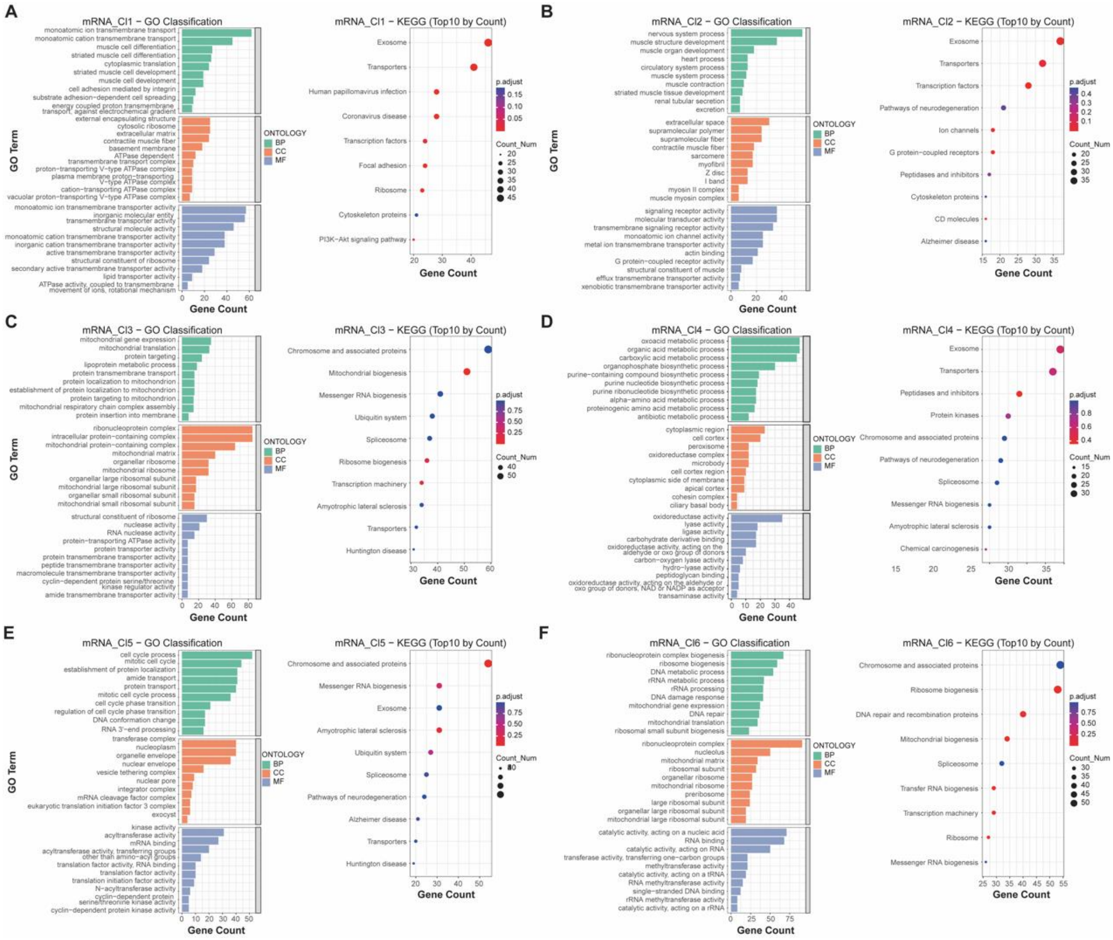

Figure S7. Functional enrichment landscape of mRNA Mfuzz clusters. (A-F) Gene Ontology (GO) terms (encompassing Biological Process, Cellular Component, and Molecular Function) and KEGG pathways enriched in Clusters 1 to 6, respectively. These clusters comprise mRNAs with significant differential expression identified via Likelihood Ratio Test (LRT). All enrichment terms are sorted in descending order based on gene count.

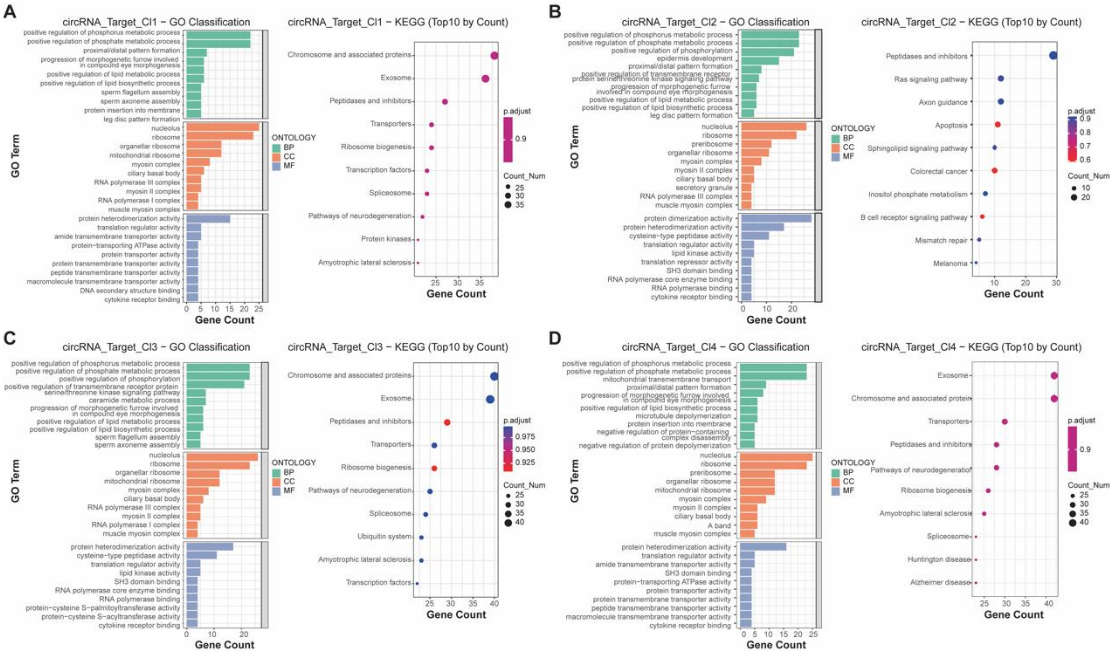

Figure S8. Functional enrichment landscape of target genes for lncRNA Mfuzz clusters. (A-D) GO terms

(encompassing Biological Process, Cellular Component, and Molecular Function) and KEGG pathways enriched for the predicted target mRNAs of LRT-significant lncRNAs across Clusters 1 to 4. Enrichment terms are sorted in descending order of target gene count.

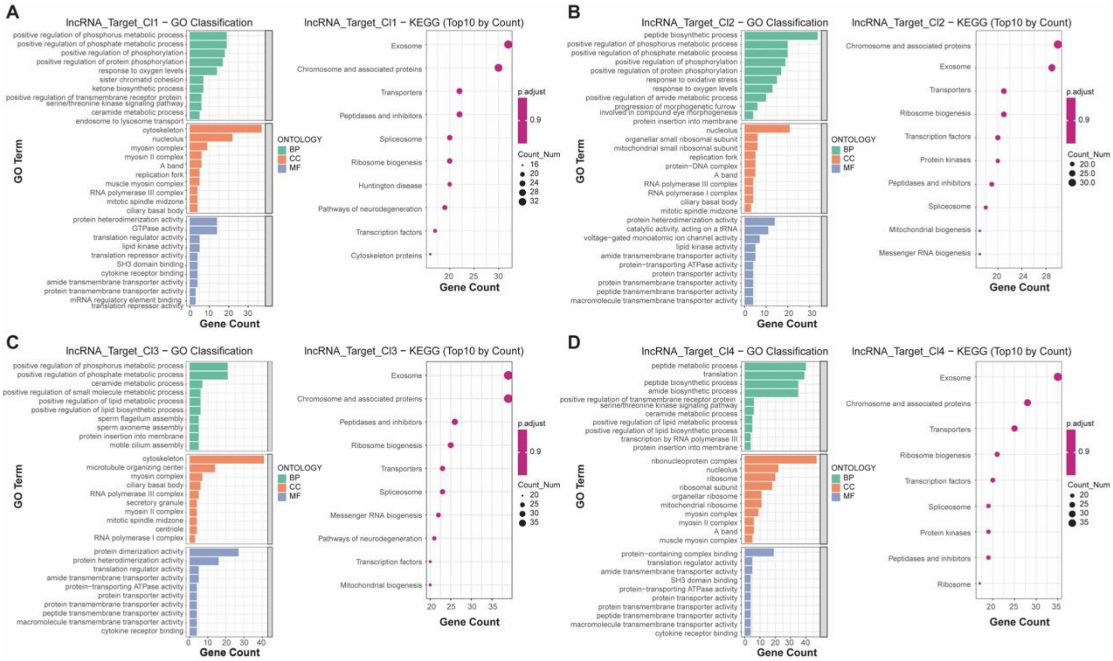

Figure S9. Functional enrichment landscape of target genes for circRNA Mfuzz clusters. (A-D) GO terms (encompassing Biological Process, Cellular Component, and Molecular Function) and KEGG pathways enriched for the predicted target mRNAs of LRT-significant circRNAs across Clusters 1 to 4. Enrichment terms are sorted in descending order of target gene count.

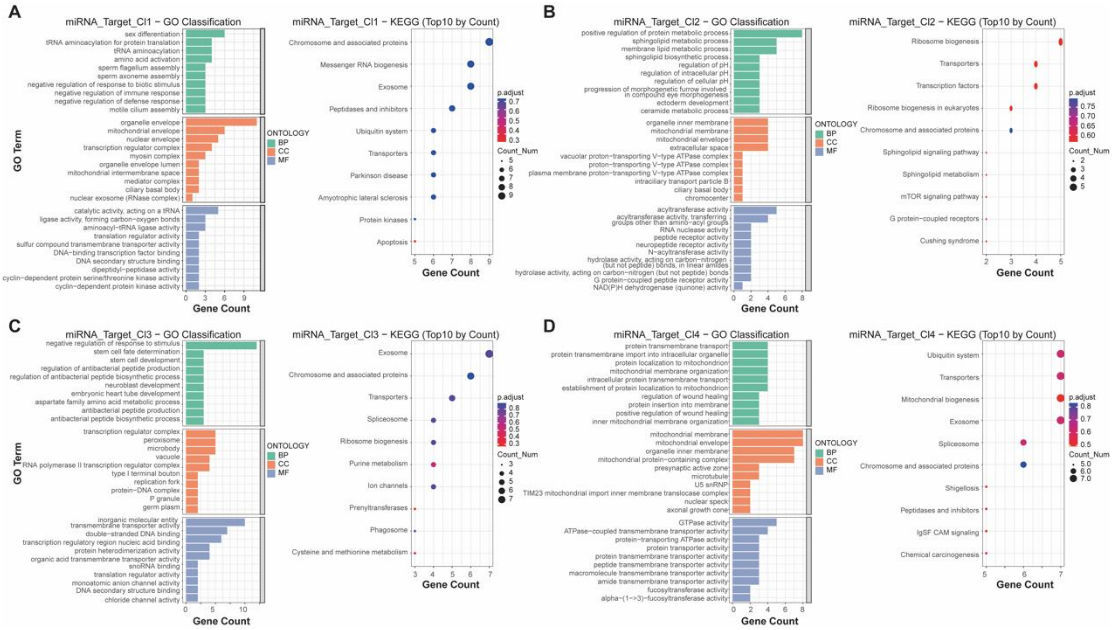

Figure S10. Functional enrichment landscape of target genes for miRNA Mfuzz clusters. (A-D) GO terms (encompassing Biological Process, Cellular Component, and Molecular Function) and KEGG pathways enriched for the predicted target mRNAs of LRT-significant miRNAs across Clusters 1 to 4. Enrichment terms are sorted in descending order of target gene count.

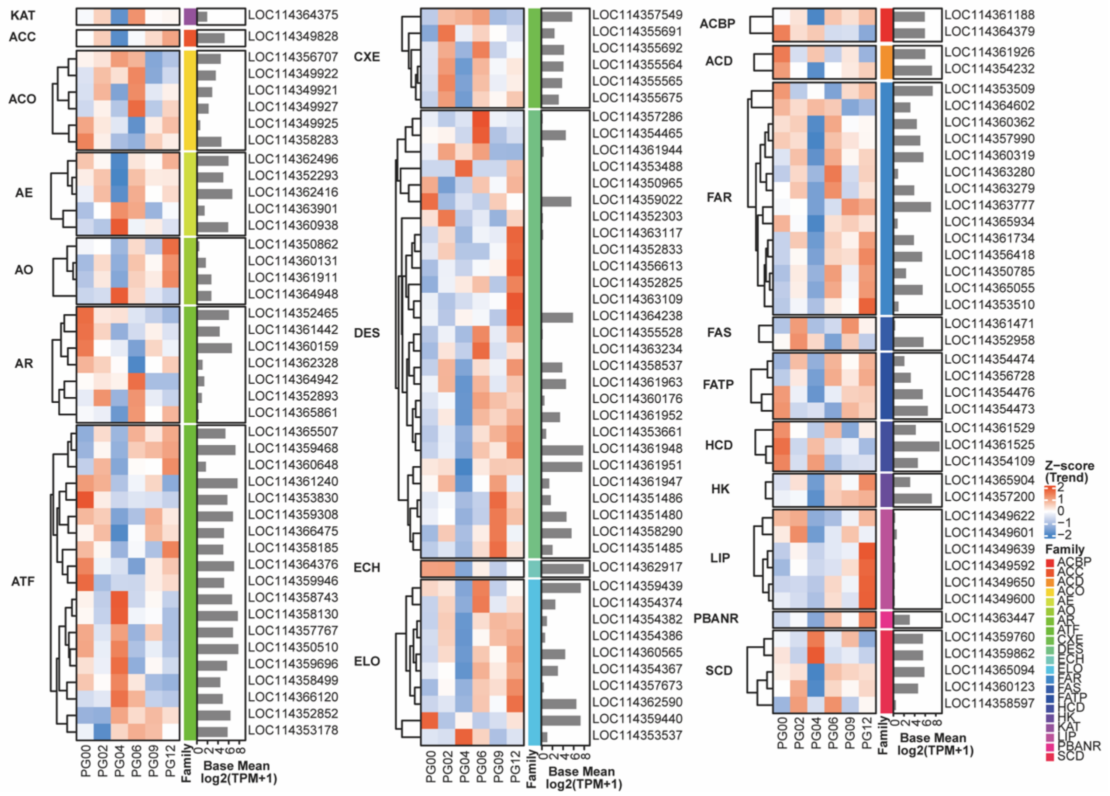

Figure S11. Temporal expression dynamics and absolute abundance of putative pheromone biosynthesis gene families. The heatmap visualizes the relative expression patterns (normalized by Row Z-score) of candidate genes across time points post-PBAN injection (PG00 to PG12). Rows are faceted by gene families potentially involved in pheromone biosynthesis and hierarchically clustered within each facet based on Euclidean distance. The right-side annotation barplot quantifies the global base mean transcript abundance, calculated as log2(TPM+1), for each gene across all temporal samples.

Table S1. All primers used in this study.

Gene	Forward primer
DES/LOC114358537	TGACAAGCCAGAGGAC
FAR/LOC114357990	AAACAAGCTTGAAAAGGCCG
PBANR/LOC114363447	CTGTGAAGGGTCAGGGAGTG
ACC/LOC114349828	GGAAACGAGGACGGTAG
ofu-circ-0325	CGTTTCCTTCTCCGATATTGTG
ofu-circ-2560	TCTTCTAACAGCCACTCTATCC
lncMSTRG.13128	CTCCCTACGCCTCAGGTAAG
lncMSTRG.21768	CCGGCCAGTATTGTGAATCG
β-actin	CCTCCACCTCCTCGAGAAG
U6	CTCGCTTCGGCAGCACA
miR-2767	CAAGTAAATCTCGTGCGG
miR-193	GTACTGGCCTGCTAAGTC

Table S2. RNA-seq clean data quality inspection analysis.

Exon_Tags	Exon_Rate(%)	Intron_Tags	Intron_Rate(%)	Intergenic_Tags
51164903	74.7	4879430	7.12	12451941
49355986	75.64	4511387	6.91	11384396
47982637	76.1	5158928	8.18	9912409
60060373	69.58	9472968	10.97	16785644
43978145	68.61	6246045	9.74	13872982
48921770	75.45	4521909	6.97	11392727
52885713	72.97	5268144	7.27	14319193
50132415	73.61	4821022	7.08	13155796
52428378	73.65	5011152	7.04	13747544
47448430	72.18	6394992	9.73	11893323
52823538	75.72	5748053	8.24	11191946
46395989	69.07	7715035	11.49	13062504

Table S3. miRNA-seq clean data quality inspection analysis.

Sample	Total_Input	High_Quality(Clean)	3_Adapter_Null	Insert_Null(Too_Short)	Mapped_to_miRNA_Reads
PG00-A	14,967,389	13,358,663	1,082,722	485,915	1,339,525
PG00-B	11,867,303	10,456,736	616,084	442,793	988,620
PG00-C	14,263,367	13,409,971	535,312	292,253	2,242,201
PG02-A	14,484,568	13,133,331	467,002	845,026	2,579,043
PG02-B	10,092,670	9,182,989	516,706	284,635	1,407,982
PG02-C	16,155,269	14,947,257	649,596	353,553	1,892,389
PG04-A	16,512,111	15,238,443	709,615	329,210	1,019,242
PG04-B	17,081,352	15,879,350	712,415	338,385	1,060,547
PG04-C	14,878,116	13,957,445	604,889	283,300	1,023,569
PG06-A	14,254,931	13,128,144	811,889	261,654	1,723,524
PG06-B	14,656,275	13,733,053	586,994	309,607	1,930,009
PG06-C	10,311,849	9,148,638	449,062	288,241	1,283,242
PG09-A	12,488,639	11,373,219	468,625	614,668	1,309,393
PG09-B	13,405,243	12,106,198	554,283	569,448	1,211,080
PG09-C	15,567,516	14,120,920	679,225	557,672	1,776,325
PG12-A	14,551,881	13,447,872	461,895	544,774	1,336,006
PG12-B	15,288,030	13,639,876	612,291	977,448	1,119,418
PG12-C	14,410,406	13,093,201	492,756	669,949	1,227,405
